# Patterns of bone marrow aspiration confirmed hematological malignancies in Eritrean National Health Laboratory

**DOI:** 10.1186/s12878-019-0138-3

**Published:** 2019-05-02

**Authors:** Natnael Belai, Amon Solomon Ghebrenegus, Anim Ata Alamin, Ghirmay Embaye, Amanuel Kidane Andegiorgish

**Affiliations:** 1 Orotta National Medical Surgical Referral Teaching Hospital, Asmara, Eritrea; 2 Ghindae Zonal Referral Hospital, Ghindae, Eritrea; 3 Department of Pathology, University of taif, Taif, Saudi Arabia; 4 National Health Laboratory, Asmara, Eritrea; 5 Asmara Collage of Health Science, Asmara, Eritrea

**Keywords:** Hematologic malignancies, Bone marrow aspiration, Distribution, Eritrea

## Abstract

**Background:**

Even though the incidences of hematologic malignancies have received considerable attentions globally, there is paucity of information on patterns of hematologic malignancy in Eritrea. The study was conducted to determine the distribution of various hematologic malignancies among patients who have received bone marrow examination, in the Eritrean National Health Laboratory.

**Methods:**

A retrospective descriptive study design was used to determine the patterns of Hematologic malignancies diagnosed at the Eritrean National Health Laboratory from October 2015 to July 2017.

**Results:**

Out of 207 patients who did bone marrow aspiration 52 patients were hematologic malignancy cases. The male to female ratio was 1:1. The age of the patients ranged from 2 to 80 years. Of the 52 patients 19, were less than 20 years of age and the remaining 33 were 20 years and above. Acute leukemia was the most common hematologic malignancy in the study area. It affected 18 of the cases followed by chronic myelogenous leukemia, chronic lymphocytic leukemia, myelodysplastic syndromes, multiple myeloma, and myeloprofilerative neoplasms. The presenting signs and symptoms in their decreasing frequency were generalized body weakness/fatigue, splenomegaly, fever, anemia, and lymphadenopathy. More than two-third of the patients had total leukocyte count greater than 10,000/μl.

**Conclusion:**

This study shows that hematologic malignancies are not uncommon in Eritrea.

## Background

Cancer is a group of diseases characterized by uncontrolled growth and spread abnormal cells [1]. Cancer is increasingly recognized as the critical public health problem in Africa. Despite the growing burden, cancer is receiving low attention by policy makers, especially in developing countries. This could be due to either limited resources, the burdens of communicable diseases and other pressing public health problems, or it may be due to lack of awareness about the magnitude and burden of the diseases both at the present and future [2]. Eritrea is a country in east Africa experiencing an annual increase in the incidence of cancer. The age standardized rate for all cancers in Eritrean hospitals and Eritrean National Health Laboratory from 2000 to 2010 was 20.3 per 100,000 [3].

Hematologic malignancies (HM) are a group of cancers that arise from a malignant transformation of cells of the bone marrow or the lymphatic system [4]. According to the study by Ferlay J et al., 2014, HMs were estimated to represent about 6.5% of all cancers worldwide in 2012 [5]. Different etiological factors are believed to contribute to the development of these HMs as their incidence varies with geography, age and race/ethnicity. Even though environmental exposure to chemicals (such as pesticides, benzene, smoking etc.), as well as ionizing radiation and infectious agents are believed to be the causes of those malignancies, their exact cause remains unclear [6, 7]. There has been a considerable rise in the occurrence of HM over the past decades. World health organization predicts the number of HM cases to increase by about 48% in less developed countries in 2030 when compared to 2012 [5].

HMs are major burdens to afflicted patients and their families psychologically, medically and financially. As to our knowledge, no study has investigated the distribution of HM in Eritrea. It also remains a mystery whether the pattern of these malignancies follows a similar course to those reported in other countries. The aim of this research is therefore to describe the pattern of bone marrow aspiration confirmed HM in Eritrea based on age, gender, most common presenting signs and symptoms and the pattern of the complete blood cell count.

## Methods

### Study design

A retrospective study design was used to analyse the data recorded in Eritrean National Health Laboratory (ENHL) from October 2015 to July 2017.

### Study population

The study population comprises of all patients who underwent bone marrow aspiration in the ENHL during the study period. The ENHL is the only laboratory in Eritrea which is capable of carrying out such investigations. The study population includes patients that were referred from different health facilities across the country for analyses. All data from the registration book was checked. Age, gender, address and most common presenting symptoms or complaints and automated complete blood count of each patient were recorded.

### Procedure of bone marrow aspiration

Patients who were clinically suspected of having HMs, visceral leishmaniasis and pancytopenia were further tested with bone marrow aspiration. Aspiration was drawn from the iliac bone and Wright and Giemsa staining was done to the drawn samples followed by microscopic examination by a licenced hemopathologist.

### Data analysis

Data was entered in excel sheet. Tables were used for presentation of the data. Percentages of the various categories of HMs were presented. The relationship between various HMs and demographic variables were explored using percentages in tables.

### Ethical clearance

Ethical approval was sought out from the Research Ethics and Protocol Review Committee of the Ministry of Health of Eritrea in Asmara, Eritrea. Approval letter to proceed was sent to the Office to the National Health Laboratory. All concerned personnel of the National Health Laboratory were briefed on the objectives of the study. Confidentiality was assured before the study and hence data use was permitted for study purpose only.

### Exclusion criteria

Patients registration/files found with incomplete information on variables like gender, age, and diagnosis were not included in this study.

## Result

A total of 207 patients had undergone bone marrow aspiration examination from October 2015to July 2017. Out of these patients, 52 were diagnosed with HM. The male to female ratio of HM was 1:1. (Table 1). Age distribution of study subjects ranged from 2 to 80 years. (Table 1) Acute leukaemia (AL) was the most prevalent HM in this study. It affected 18 (34.6%) of the cases followed by chronic myeloid leukemia (CML) (28.8%), chronic lymphocytic leukaemia (CLL) (23.1%), myelodysplastic syndrome (MDS) (7.7%), Multiple Myeloma (MM) (3.8%), and myeloproliferative neoplasm (MPN) (1.9%).

**Table 1:** Distribution of HMs according to gender and age

Type of HM	Gender			Age group (in years)			
	Male	Female	0–20	21–40	41–60	61–80	Total
AL	10	8	14	3	1	0	18
CLL	7	5	1	0	6	5	12
CML	7	8	3	4	7	1	15
MDS	0	4	1	1	2	0	4
MPN	0	1	0	0	0	1	1
MM	2	0	0	0	1	1	2

*Abbreviation*: *HM* hematologic malignancy, *AL* acute leukemia, *CLL* chronic lymphocytic leukaemia, *CML* chronic myeloid leukemia, *MDS* myelodysplastic syndrome, *MM* Multiple Myeloma, *MPN* myeloproliferative neoplasm

Patients also had different clinical presentations and were categorized as: Generalized body weakness (GBW)/fatigue; Splenomegaly; Fever; Anemia; Lymphadenopathy; Weight loss/ loss of appetite; Headache/light headedness/blurred vision/dizziness; Left upper quadrant (LUQ) pressure/distension; Bleeding diatheses; Shortness of breath (SOB)/cough/respiratory distress; Hepatomegaly; Joint pain/swelling; and Others (sore throat, dyspepsia, diarrhea, vomiting, hypercalcemia, hypoalbuminemia).

Around one-fourth (26.7%) of patients with CML and one-sixth (16.7%) of CLL presented with LUQ pressure sensation or swelling. Around three-fourth (74%) of the patients with CML also presented with splenomegaly followed by AL (39%) and CLL (9%). The patients with AL, CLL, CML and MDS presented with GBW or fatigue with 44.4, 41.7, 33.3 and 75% respectively. Hepatomegaly was present in 11% of the patients with AL and 13.3% of patients with CML. Lymphadenopathy was seen in 16.7% of patients with AL and 50% of patients with CLL. Patients with AL, CLL and CML presented with fever 44.4, 41.7 and 26.7% respectively. Anemia was common to all HM except for MPN (Table 2).

**Table 2:** Distribution of Hematologic Malignancies by clinical presentation

Variables	Hematologic Malignancies [Number]						Total
	AL	CLL	CML	MDS	MPN	MM	
GBW/fatigue	8	5	5	3	0	0	21
Splenomegaly	7	1	11	0	0	0	19
Fever	8	5	4	0	0	0	17
Anemia	5	2	1	1	0	1	10
Lymphadenopathy	3	6	0	0	0	0	9
Weight Loss	1	3	3	0	0	0	7
Headache	3	1	3	0	0	0	7
Bleeding diathesis	4	0	1	1	0	0	6
LUQ distention	0	2	4	0	0	0	6
Shortness of breath	4	0	0	1	0	0	5
Hepatomegaly	2	0	2	0	0	0	4
Joint pain	3	0	0	0	0	0	3

*Abbreviation*: *GBW* Generalized Body Weakness, *LUQ* Left Upper Quadrant

Forty-eight (92.3%) of the 52 patients who underwent bone marrow aspiration had results of complete blood cell count. Out of these 33 had total WBC count greater than 10,000/μl. 42 had hemoglobin level less than 12 g/dl. 28 had MCV between 85 and 100 fL and 13 had MCV greater than 100 fL. 29 of the patients had platelet count less than 150,000/μl and about 5 had platelet counts greater than 450,000/μl (Table 3).

**Table 3:** Distribution of HM according to Complete Blood Count

Variable	Value	Hematologic Malignancies [Number]						Total [Number]
		AL	CLL	CML	MDS	MPN	MM	
WBC (× 10^3^/μl)	> 10	8	11	14	0	0	0	33
	< 10	8	1	0	4	1	1	15
HGB (g/dl)	< 12	16	8	13	4	0	1	42
	> 12	0	4	1	0	1	0	6
MCV^a^ (fL)	> 100	4	4	4	0	1	0	13
	85–100	9	7	8	3	0	1	28
	< 85	3	1	2	0	0	0	6
PLT (×10^3^/μl)	> 450	0	0	4	0	1	0	5
	150–450	0	4	8	1	0	1	14
	< 150	16	8	2	3	0	0	29

*Abbreviation*: *WBC* White blood cells, *HGB* Hemoglobin, *MCV* Mean cell volume, *PLT* Platelets

^a^ One patient’s MCV value was missing

## Discussion

To our knowledge this is the first study in Eritrea that describes the pattern and distribution of HM in terms of age, gender, common clinical presentation and the pattern of the CBC.

AL was the most common HM and accounted for 18 of the 52 HMs, whereas CML and CLL were the second and third most recurring HMs, accounting for 15 and 12 of the cases respectively. This was consistent with the findings of studies from Bangladesh and Iran [8, 9]. Studies carried out in Yemen and Nigeria reported AL to be the third most common HM [10, 11]. Studies in Eastern Morocco, Egypt, South Africa, Yemen and Nigeria all conclude as NHL as their most common HM. Other than the South African study, all the above mentioned studies also report HL as their second most recurring HM [8, 10–13].

Of the 52 cases, HMs were most commonly diagnosed in ages less than 20 years, with 19 cases, followed by the 40–60 age group. Similarly, ALs were more common in the age group-under 20 years making up 14 of the patients, while CML was found most common in patients over 20 years (12), followed by CLL (11), MDS (3), MM (2) and MPN (1). Compared to the previous studies, the findings of the present study were found to be inconsistent. For example, Eastern Morocco reported that the most common HM in patients less than 20 years of age were the lymphomas [10]. The study also reported that the most HMs were diagnosed in patients aged greater than 60 years [10]. Hossain et al. also had similar findings [11]. We suspect that this may be due to our inability to include the lymphomas in this study.

The presenting signs and symptoms of the present study in their descending percent were GBW/fatigue; Splenomegaly; Fever; anemia; Lymphadenopathy; Weight loss/ loss of appetite; Headache/light headedness/blurred vision/dizziness. This was in line to the findings of studies done by Prajapati et al., Weldetsadik AT, Das et al. and Siddaiahgari et al. [14–17]. Majority (42) of the patients with HM were found to be anemic in our study.

In the present study the male to female ratio was 1:1. Many studies carried out in the Middle East, Asia, Africa and the UK reported a male predominance in HMs ranging from 1.1 (Eastern Morocco) to 2.2 (Bangladesh) [8–12, 18, 19].

The study has a number of limitations. As there is no functioning histopathology unit in Eritrea, we were forced to exclude HMs that require tissue biopsy like lymphomas and only analyzed patients who underwent bone marrow aspiration to confirm the HMs. The other limitation is the nature of the study. As it is retrospective data analysis, many of the results had not distinguished ALL from AML, labeling them both as AL. Therefore, we had to combine ALL and AML to AL. The morphological analysis of the samples and the absence of detailed studies like flow cytometry or cytogenetic studies along with the small sample size are also important limitations of the study.

## Conclusion

This study elaborates that HMs are present in Eritrea. This study was the very first step in understanding the patterns and distribution of these diseases. Further inquiries are needed to clearly establish the epidemiology, potential risk factors, biology and genetics of HMs in Eritrea. Moreover, physicians should be aware that peripheral blood smears could also be used to increase suspicion of the disease.

## Abbreviations

AL: Acute leukemia
ALL: Acute lymphoblastic leukemia
AML: Acute myeloid leukemia
CLL: Chronic lymphocytic leukemia
ENHL: Eritrean National Health Laboratory
GBW: Generalized Body weakness
HGB: Hemoglobin
HL: Hodgkin’s lymphoma
HM: Hematological malignancies or Hematological malignancy
LUQ: Left upper quadrant
MCV: Mean cell volume
MDS: Myelodysplastic syndromes
MM: Multiple myeloma
MPN: myeloprofilerative neoplasms
NHL: Non-Hodgkin’s lymphoma
PLT: Platelets
SOB: Shortness of breath
WBC: White blood cells
