# Correction to: Patterns of bone marrow aspiration confirmed hematological malignancies in Eritrean National Health Laboratory

**DOI:** 10.1186/s12878-019-0143-6

**Published:** 2019-05-31

**Authors:** Natnael Belai, Amon Solomon Ghebrenegus, Amin Ata Alamin, Ghirmay Embaye, Amanuel Kidane Andegiorgish

**Affiliations:** 1 Orotta National Medical Surgical Referral Teaching Hospital, Asmara, Eritrea; 2 Ghindae Zonal Referral Hospital, Ghindae, Eritrea; 3 Department of Pathology, University of taif, Taif, Saudi Arabia; 4 National Health Laboratory, Asmara, Eritrea; 5 Asmara Collage of Health Science, Asmara, Eritrea

**Correction to: BMC Hematol**

**https://doi.org/10.1186/s12878-019-0138-3**

The original version of this article [1] unfortunately included an error to an author’s name. Author Amin Ata Alamin was incorrectly presented as Anim Ata Alamin.

The correct author name has been included in the author list of this Correction article and is already updated in the original article.
